# Wrist Arthrodesis in Rheumatoid Arthritis Using an LCP Metaphyseal Locking Plate versus an AO Wrist Fusion Plate

**DOI:** 10.1155/2018/4719634

**Published:** 2018-07-10

**Authors:** Toshitake Taii, Takumi Matsumoto, Sakae Tanaka, Ichiro Nakamura, Katsumi Ito, Takuo Juji

**Affiliations:** ^1^Department of Rheumatology, JCHO Yugawara Hospital, 438 Miyakami, Yugawara, Ashigara-shimo, Kanagawa 259-0396, Japan; ^2^Department of Orthopaedic Surgery, Faculty of Medicine, The University of Tokyo, 7-3-1 Hongo, Bunkyo-ku, Tokyo 113-8655, Japan; ^3^Faculty of Medical Science for Health, Teikyo Heisei University, 2-51-4 Higashi-Ikebukuro, Toshima, Tokyo 170-8445, Japan; ^4^Department of Orthopaedic Surgery, Heisei Yokohama Hospital, 550 Totsuka, Totsuka-ku, Yokohama, Kanagawa 244-0003, Japan

## Abstract

**Objectives:**

Although wrist arthrodesis using a plate is an established treatment with a well-documented successful union rate for severely destroyed wrists, plate-related complications are a matter of great concern.

**Methods:**

We retrospectively compared wrist arthrodesis using an AO wrist fusion plate in nine and a locking compression plate (LCP) metaphyseal plate in seven cases of rheumatoid arthritis.

**Results:**

The mean follow-up was 40.6 months in the AO wrist fusion plate group and 57.2 months in the LCP metaphyseal plate group. Bone union at the arthrodesis site was achieved in all cases in both groups. Comparison of the original position of the fusion on the immediate postoperative radiographs and the position on the most recent follow-up radiographs demonstrated good stability in both groups. Plate-related complications occurred in four cases in the AO wrist fusion plate group and no cases in the LCP metaphyseal plate group. Complications included pain over the plate, wound dehiscence and infection, extensor tendon adhesion, and fracture in one case each.

**Conclusion:**

Wrist arthrodesis using an LCP metaphyseal plate was favorable for rheumatoid arthritis patients with comparable stability to that of and a lower risk of plate-related complications than an AO wrist fusion plate.

## 1. Introduction

Rheumatoid arthritis (RA) is a systemic autoimmune disease characterized by inflammation of the synovial membranes within the joint. The wrist is the most commonly involved joint from early in the disease course and shows the most severe progression of all joints in patients with RA [1]. A long-term follow-up study demonstrated that 75% of patients had erosive wrist disease and nearly 40% of wrist joints were already fused or showed severe destruction over 15–20 years [2]. Early changes in the wrist joint can be managed by surgical methods such as synovectomy, arthroplasty, and partial wrist fusion; however, total wrist arthrodesis is the gold standard and a well-established surgical treatment for severely destroyed RA wrists, providing a satisfaction rate >90% [3].

Several methods have been described for arthrodesis of the RA wrist, including bone graft and immobilization [4], intramedullary pins supplemented with staples or Kirschner wires [5, 6], and plates [7]. Good primary stability achieved by the dynamic compression plate have made its use for wrist arthrodesis most popular [7]. On the other hand, plate fixation has the disadvantage of hardware prominence that causes extensor tendon irritation or skin breakdown, which often requires plate removal. Although use of an AO plate has been popular for wrist fusion since its introduction by Heim and Pfeiffer in 1974 [8], it is reportedly accompanied by plate-related complications requiring implant removal in around 10% of cases [7].

Various locking plates are now available owing to their clinical success, and surgeons now have more device choices. As a preventive measure for decreasing plate-related complications, we first adopted the locking compression plate (LCP) metaphyseal plate for RA wrist arthrodesis instead of an AO wrist fusion plate in 2012. Here, we report our experience in seven patients with RA who underwent total wrist arthrodesis using an LCP metaphyseal plate versus nine patients with RA treated with an AO wrist fusion plate.

## 2. Patients and Methods

### 2.1. Patients

**Figure 1:**
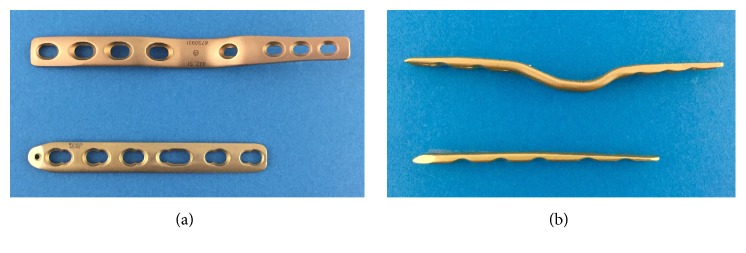
Photographs showing the AO wrist fusion plate with a short bend and the LCP metaphyseal plate 3.5 from top view (a) and side view (b).

**Figure 2:**
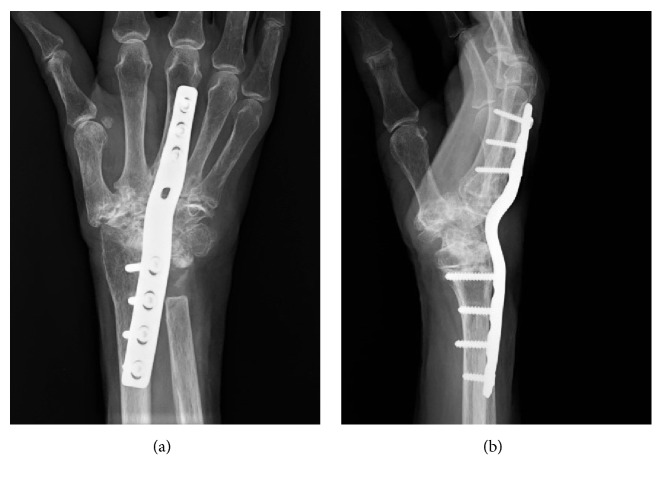
Representative anteroposterior (a) and lateral (b) radiographs of wrist arthrodesis using the AO wrist fusion plate.

**Figure 3:**
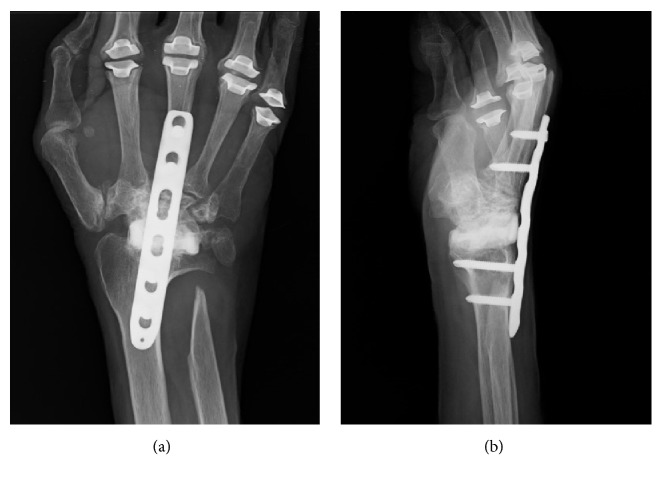
Representative anteroposterior (a) and lateral (b) radiographs of wrist arthrodesis using LCP metaphyseal plate.

We retrospectively analyzed 16 consecutive cases (13 patients with RA) in which arthrodesis of the wrist was performed between August 2001 and March 2016. The implants used for wrist arthrodesis were the AO wrist fusion plate (DePuy Synthes, Tokyo, Japan) in nine cases before 2011 (Figures 1 and 2) and the LCP metaphyseal plate 3.5 (DePuy Synthes) in seven cases after 2012 (Figures 1 and 3). All procedures were performed at JCHO Yugawara Hospital (Kanagawa, Japan) by the senior doctors (K.I. and T.J.) or by the attending surgeons under supervision of the senior doctors. All patients signed a consent form and the study was approved by our institution's ethical committee.

### 2.2. Clinical Assessments

The medical charts, radiographs, and operative records of each patient were referenced to investigate the postoperative complications, bone union, loss of correction, implant size, number of screws, and additional procedures performed at the same time as the wrist arthrodesis. Bone union was defined as radiographic trabecular continuity at the arthrodesis site, whereas clinical stability was determined by the absence of instability and tenderness on pressure. The ulnar deviation angle was measured on the anteroposterior radiographs of the hands and wrists as the angle between the long axis of the radius and that of the third metacarpal. The wrist extension angle was measured on the lateral radiographs of wrists as the angle between the long axis of the radius and that of the third metacarpal. These measurements were performed on the radiographs immediately after surgery and at the most recent follow-up, and the differences between the time points were evaluated as an index of stability after the plate fixation. The distance from the metacarpal head to the distal end of the plate along the axis of the third metacarpal was measured on the immediate postoperative radiographs in all cases except those treated with implant arthroplasty at the third metacarpophalangeal joint. For these cases, the difference between the length of the third metacarpal in the preoperative radiograph and the length of the plate at the metacarpal on the immediate postoperative radiograph was calculated instead. An independent observer (board certified member of the Japanese Orthopedic Association and Japan College of Rheumatology) assessed the patient's clinical and radiographic progress to minimize interobserver bias.

### 2.3. Operative Technique

A midline dorsal longitudinal incision was made over the wrist from the midshaft of the third metacarpal to the distal radius. After the extensor retinaculum was opened over the fourth extensor compartment and a synovectomy of the extensor tendon was performed, the joint capsule was divided longitudinally and the carpal bones were exposed. After the cartilage and subchondral bone were removed from the remaining articular surfaces within the wrist, the bone graft was packed into the clearance gaps. The source of the bone graft included the resected distal ulna, distal radius, and resected carpal bones, and an allograft was also used in three cases in the AO wrist fusion plate group and five cases in the LCP metaphyseal plate group.

A plate extending from the third metacarpal to the radius was used for wrist fixation. The position of the fused wrist was targeted at 10–20 degrees of extension and 5–10 degrees of ulnar deviation, which are thought to be preferable for patient satisfaction and the prevention of further ulnar deviation of the phalanges [9–11]. A slight bend was applied in the plate to contour the targeted wrist position. Eighteen concurrent operative procedures were performed in the 11 cases. These procedures included finger metacarpophalangeal joint arthroplasty using a Swanson silicone implant in five cases, arthrodesis of thumb metacarpophalangeal or interphalangeal joints in four, arthrodesis of finger proximal interphalangeal or distal interphalangeal joints in four, extensor tendon transfer in three, and synovectomy of the finger metacarpophalangeal joint in two cases. Postoperative immobilization was provided by a forearm splint for 2 weeks.

### 2.4. Statistics

All continuous variables are reported as mean (range). Radiographic measurements of the two groups were compared using repeated-measures analysis of variance and post hoc Tukey's honest significant difference tests. Paired t-tests were used to assess changes in the radiographic measurements from immediately after surgery to the most recent follow-up. Statistical analyses of the data were performed using JMP12 statistical software (SAS Institute Inc., Cary, NC, USA).

## 3. Results

The patients' backgrounds and clinical assessments other than radiographic measurements in the AO wrist fusion group and the LCP metaphyseal plate group are shown in Tables 1 and 2, respectively. Three patients underwent bilateral wrist arthrodesis; two patients received one AO wrist fusion plate and one LCP metaphyseal plate, while one patient had both sides using LCP metaphyseal plate. The nine patients in the AO wrist fusion plate group (eight women, one man) had a mean age of 57.3 years (range, 28–81 years), while the seven patients in LCP metaphyseal plate group (five women, two men) had a mean age of 64.1 years (range, 60–72 years). The mean follow-up period was 57.2 months (range, 6–128 months) in the AO wrist fusion plate group and 40.6 months (range, 6–65 months) in the LCP metaphyseal plate group.

Successful union was achieved within 6 months in all cases.

The plate used in the AO wrist fusion plate group was the short bend type in eight cases and the standard bend type in one case; no straight type plates were used. A mean of 7.3 screws (range, 7-8 screws) were used per plate for a mean of 3.6 screws (range, 3–4 screws) in the proximal aspect of the third metacarpal and a mean of 3.8 screws (range, 3–4 screws) in the distal radius. The most common plate length used in the LCP metaphyseal plate group was six holes in four cases, followed by seven holes in two cases and eight holes in one case. A mean of 5.1 screws (range, 4–6 screws) were used per plate for a mean of 2.1 screws (range, 2–3 screws) in the proximal aspect of the third metacarpal and a mean of 3.0 screws (range, 2–4 screws) in the distal radius. No screws were placed into the carpal bones in our series.

The radiographic measurement results are shown in Table 3. Wrists in the AO wrist fusion plate group averaged 13.9 degrees of extension (range, 10–18 degrees) and 7.2 degrees of ulnar deviation (range, 2–15 degrees) immediately after surgery and 14.4 degrees of extension (range, 10–18 degrees) and 8.7 degrees of ulnar deviation (range, 2–15 degrees) at the most recent follow-up. Both wrist extension angle and ulnar deviation in the AO wrist fusion plate group did not differ significantly between immediately after surgery and the most recent follow-up (*p* = 0.72,* p* = 0.11, respectively). Wrists in the LCP metaphyseal plate group averaged 21.4 degrees of extension (range, 16–24 degrees) and 12.3 degrees of ulnar deviation (range, 3–21 degrees) immediately after surgery and 20.6 degrees of extension (range, 16–27 degrees) and 12.3 degrees of ulnar deviation (range, 7–20 degrees) at the most recent follow-up. Both wrist extension angle and ulnar deviation in the LCP metaphyseal plate group showed no significant difference between immediately after surgery and the most recent follow-up (*p* = 0.67,* p* > 0.99, respectively). The absolute value of the change from immediately after surgery to the most recent follow-up was not significantly different between the AO wrist fusion plate and LCP metaphyseal plate groups for both wrist extension angle (3.0 degrees [range, 0–5 degrees] versus 3.6 degrees [range, 2–6 degrees],* p* = 0.57) and ulnar deviation (1.8 degrees [range, 0–5 degrees] versus 3.0 degrees [range, 0–5 degrees],* p* = 0.28). These comparisons showed that the AO wrist fusion and LCP metaphyseal plates provided similar bone stability in patients with RA. The distance between from the third metacarpal head to the distal end of the plate was significantly greater in the metaphyseal plate group than in the AO wrist fusion plate group (2.8 cm [range, 2.5–3.4 cm] versus 2.1 cm [range, 1.5–3.2 cm],* p* = 0.005).

Four complications occurred in the AO wrist fusion plate group versus no complications in the LCP metaphyseal plate group. The complications were as follows: (1) tenderness over the plate, (2) wound dehiscence and infection, (3) adhesions of the extensor tendon, and (4) fracture of the radius at the proximal end of the plate (case nos. 4, 5, 7, and 8, respectively). All four cases required plate removal for resolution.

## 4. Discussion

The present study demonstrated that, similar to the AO wrist fusion plate, the LCP metaphyseal plate could provide sufficient stability for wrist arthrodesis in RA patients with fewer plate-related complications.

Since its development, the dynamic compression plate has become the most popular method for arthrodesis of the wrist destroyed by various pathologies. The use of a 3.5-mm dynamic compression plate was initially popular for wrist arthrodesis; however, it was accompanied by complications in a reported 51% of cases [12–16]. Postoperative complications were mainly plate-related and included plate prominence; skin irritation; wound dehiscence and infection; rupture, adhesion, and tenosynovitis of the extensor tendon; and fracture at a plate hole [17]. Synovitis of the extensor tendon and pain at the distal end of the plate are recognized as one of the major complications [13, 16]. This led to the introduction of a tapered AO wrist fusion plate, which has a tapered end tailored to the metacarpal shaft, 3.5-mm screws proximally, and 2.7-mm screws distally [18].

Despite this refinement, the AO wrist fusion plate has not been free of plate-related complications [7, 19, 20]. The largest series of wrist arthrodesis with an AO wrist fusion plate, which included 42 posttraumatic arthritis patients and three RA patients, reported highly satisfactory functional results but six cases (14%) of plate-related complications requiring plate removal [19]. Another study of 17 wrist arthrodesis procedures with AO wrist fusion plate including two RA patients reported successful bone union and a high patient satisfaction rate but two cases of plate-related complications (12%): one of extensor tendinitis and one of fracture through a screw hole [20]. The comparative study of wrist arthrodesis performed using the Mannerfelt technique and that with an AO wrist fusion plate in RA patients reported significantly greater satisfaction with wrist function in the AO wrist fusion plate group but six complications in 23 patients (26%) treated with the AO wrist fusion plate [7]. Complications included one superficial infection, two deep infections requiring plate removal, one postoperative extensor tendon rupture, one case of hypoesthesia of the digits, and one case of plate breakage. In our present study including nine cases of wrist arthrodesis in RA patients with an AO wrist fusion plate, plate-related complications requiring plate removal occurred in four cases (44%): pain over the plate, wound dehiscence and infection, extensor tendon adhesion, and fracture in one case each. The high occurrence of complications in the present study compared with other studies is presumably attributable to the patients in our series, all of whom had RA.

Previous reports and our own experiences using AO wrist fusion plates inspired us to identify an alternative plate with less bulk but equal fixation stability. The mechanical performance of locking plate constructs with two screws has been demonstrated to be biomechanically comparable to three non-locking screw constructs in osteoporotic bone [21]. Another biomechanical study showed that the use of two locked screws per segment provides mechanical stability equivalent to that provided by three locked screws in the osteoporotic humerus [22]. Given that a 3.5-mm dynamic compression plate and a 3.5-mm reconstruction plate showed adequate fixation with three non-locking screws in the metacarpal [16], we believed that the locking plate construct with two screws in the metacarpal would provide sufficient stability and reduce the distal plate length, where plate-related complications occur most frequently [13, 16].

The AO wrist fusion plate has two forms: a straight plate with nine screw holes and a bend plate with a curved middle and eight screw holes. A bend plate consists of standard or short bends, the difference of which is the distance from the proximal bend point to the distal end of the plate. AO wrist fusion plates have a standard width of 11 mm proximally and 8 mm distally and tapered ends with a thickness ranging from 2.9 mm to 4.1mm proximally and from 2.1 mm to 4.1 mm distally. On the other hand, the LCP metaphyseal plate 3.5, which is suggested to be used for distal fractures of the humerus, proximal fractures of the radius or ulna, and distal fractures of the fibula, has a variety of lengths with 6–12 screw holes but a standard width of 11 mm and thicknesses of 3.3 mm proximally and 1.5 mm distally. The length of the AO wrist fusion plate is 112 mm for the straight plate and 118 mm for the bend plate, while the metaphyseal plate is 86 mm for six holes, which was the commonest in our case series, 99 mm for seven holes, and 112 mm for eight holes.

In the present study, the reduced plate length at the metacarpal successfully decreased plate-related complications and provided enough space at the distal aspect of the metacarpal for arthroplasty implant insertion. In the present case series, there were three cases of silicone implant arthroplasty at the 3^rd^ metacarpophalangeal with wrist arthrodesis using an LCP metaphyseal plate. All three cases could have a silicone implant inserted without cutting short of its proximal limb. The LCP metaphyseal plate must be slightly bent to achieve the recommended wrist position of moderate extension [9–11]. Because plate bending at locking holes can compromise locking screw function [23], surgeons must avoid bending the plates at the screw holes.

This study has several limitations, including its retrospective design and small number of cases (n = 16). However, our findings on using the LCP metaphyseal plate for wrist arthrodesis are still valuable because there has not been enough evidence about this application.

In conclusion, this study's findings suggest that the LCP metaphyseal plate 3.5 is a favorable method for wrist arthrodesis in RA patients that features a comparable stability to that of and a lower risk of plate-related complications than the AO wrist fusion plate. The LCP wrist fusion plate (DePuy Synthes), which has the same configuration but being thinner than the AO wrist fusion plate and having locking screw mechanism, has been introduced to market in some regions including North America and European Union nations, and some countries in Asia-Pacific since 2008. To the best of our knowledge, there has been no literature reporting the outcomes of wrist arthrodesis using the LCP wrist fusion plate. Nonetheless, in some other countries such as Japan where the LCP wrist fusion plate is not available, we consider that the LCP metaphyseal plate 3.5 is one of the good alternatives to the AO wrist fusion plate for wrist arthrodesis in RA patients.

## Figures and Tables

**Table 1:** Data of patients in the AO wrist fusion plate group: demographics, plate type, number of screws used, postoperative complications, and follow-up period.

Case	Sex	Age (y)	Disease duration (y)	Stage	F-U period (m)	Other procedures	Bone graft	Plate type	MC screws; no.	Radial screws; no.	Complications	Plate removal
1	F	28	10	III	20	CMJ arthrodesis (I), MPJ synovectomy (III)	Auto	Short	4	4	-	-

2	M	42	9	III	19	-	Auto	Standard	4	3	-	-

3	F	58	14	IV	128	-	Auto + Allo	Short	4	4	-	-

4	F	60	24	IV	119	-	Auto + Allo	Short	3	4	Pain over the plate	YES

5	F	56	15	IV	6	IPJ arthrodesis (I)	Allo	Short	4	3	Wound dehiscence and infection	YES

6	F	52	7	IV	112	IPJ arthrodesis (I), MPJ synovectomy (III), tendon transfer for extensor tendon rupture	Auto	Short	3	4	-	-

7	F	63	23	IV	52	Tendon transfer for extensor tendon rupture	Auto	Short	3	4	Extensor tendon adhesion	YES

8	F	81	17	IV	29	-	Auto	Short	4	4	Fracture	YES

9	F	76	16	IV	30	DIPJ arthrodesis (II)	Auto	Short	3	4	-	-

M, male; F, female; y, year; m, month; Stage, Steinbrocker stage; F-U, follow-up; CMJ, carpometacarpal joint; MPJ, metacarpophalangeal joint; IPJ, interphalangeal joint; DIPJ, distal interphalangeal joint; Auto, autograft; Allo, allograft; MC, metacarpal.

**Table 2:** Data of patients in the LCP metaphyseal plate group: demographics, plate type, number of screws used, postoperative complications, and follow-up period.

Case	Sex	Age (y)	Disease duration (y)	Stage	F-U period (m)	Other procedures	Bone graft	Plate type	MC screws, no.	Radial screws, no.	Complications	Plate removal
1	F	72	22	IV	9	MPJ implant arthroplasty (II, I II, IV, V), PIPJ arthrodesis (IV), DIPJ arthrodesis (III, IV, V)	Auto + Allo	6-hole	2	3	-	-

2	F	64	28	IV	65	MPJ implant arthroplasty (II, III, IV, V)	Auto + Allo	6-hole	2	3	-	-

3	M	60	3	III	53	Tendon transfer for extensor tendon rupture	Auto	8-hole	2	4	-	-

4^*∗*^	F	21	21	IV	49	MPJ implant arthroplasty (II, III, IV, V)	Allo	6-hole	2	2	-	-

5^*∗∗*^	F	31	31	IV	51	MPJ implant arthroplasty (II, V), DIPJ arthrodesis (IV)	Auto	7-hole	2	3	-	-

6^*∗∗∗*^	M	60	3	III	51	-	Auto	7-hole	2	3	-	-

7	F	62	31	IV	6	CMJ arthrodesis (I), MPJ implant arthroplasty (III),	Auto + Allo	6-hole	3	3	-	-

M, male; F, female; y, year; m, month; F-U, follow-up; MPJ, metacarpophalangeal joint; PIPJ, proximal interphalangeal joint; DIPJ, distal interphalangeal joint; CMJ, carpometacarpal joint; Auto, autograft; Allo, allograft; MC, metacarpal

^*∗*^Contralateral side of case no. 3 in Table 1.

^*∗∗*^Contralateral side of case no. 4 in Table 1.

^*∗∗∗*^Contralateral side of case no. 3 in Table 2.

**(a):** 

	AO wrist fusion plate (n = 9)			LCP metaphyseal plate (n = 7)		
	Immediately after surgery	Most recent F-U	*P* value	Immediately after surgery	Most recent F-U	*P* value
WE angle (degrees)	13.9 (10–18)	14.4 (10–18)	0.72	21.4 (16–24)	20.6 (16–27)	0.67

Ulnar deviation angle (degrees)	7.2 (2–15)	8.7 (2–15)	0.11	12.3 (3–21)	12.3 (7–20)	> 0.99

LCP, locking compression plate; F-U, follow-up; WE, wrist extension.

**(b):** 

	AO wrist fusion plate (n = 9)	LCP metaphyseal plate (n = 7)	*P* value
|∆WE angle| (degrees)	3.0 (0–5)	3.6 (2–6)	0.57

|∆Ulnar deviation angle| (degrees)	1.8 (0–5)	3.0 (0–5)	0.28

Distance between MC head to distal end of plate (mm)	2.1 (1.5–3.2)	2.8 (2.5–3.4)	0.005

LCP, locking compression plate; WE, wrist extension; MC, metacarpal.

## Data Availability

The data will not be shared, because the data are patient data and were collected on the agreement that the individual data will not be publicly distributed.
